# Stakeholders’ perspectives on barriers and enablers of chronic kidney disease care in Ethiopia: A qualitative study

**DOI:** 10.1371/journal.pone.0336781

**Published:** 2025-11-13

**Authors:** Daniel Bekele Ketema, Min Jun, Sradha Kotwal, Workagegnehu Hailu, Martin Gallagher, Rohina Joshi

**Affiliations:** 1 . The George Institute for Global Health, Sydney, New South Wales, Australia; 2 Faculty of Medicine and Health, UNSW Sydney, Sydney, New South Wales, Australia; 3 School of Public Health, College of Medicine and Health science, Debre Markos University, Ethiopia; 4 Prince of Wales Hospital, UNSW Sydney, Sydney, Australia; 5 Department of Internal Medicine, College of Medicine and Health Sciences, University of Gondar, Ethiopia; 6 Liverpool Clinical School, UNSW Sydney, Sydney, New South Wales, Australia; 7 School of Population Health, Faculty of Medicine and Health, UNSW Sydney, Sydney, New South Wales, Australia; 8 Walter Sisulu University, Mthatha, South Africa; Chulalongkorn University Faculty of Medicine, THAILAND

## Abstract

**Background:**

Chronic kidney disease (CKD) is a growing public health problem in Ethiopia. However, evidence on the health system and contextual factors influencing CKD care remains limited. This study explored the barriers and facilitators to CKD care from the perspectives of healthcare providers and other stakeholders.

**Methods:**

A descriptive qualitative study was conducted using purposive and maximum variation sampling to recruit healthcare providers (including general practitioners, nephrologists/internists, nurse) and non-communicable disease (NCD) officers and program coordinators. Interviews were audio recorded, transcribed, and thematically analysed, underpinned by the Theoretical Domains Framework version 2.

**Results:**

Fifteen participants (six general practitioners, five nephrologists/internists, one nurse, and three NCD program officers and coordinators) were included. About 40% of participants had over six years’ experience. Key barriers to CKD care included patient misconceptions, low patient and healthcare provider awareness, shortage of health workforce, knowledge gaps among junior healthcare providers, limited resources, high out-of-pocket costs, absence of registries for CKD, weak referral systems, inconsistent access to medicines and diagnostics, lack of structured training, and conflict-related disruptions. Facilitators included adherence to guidelines by senior staff, inclusion of CKD into national non-communicable disease strategies, and increased use of media for public health education.

**Conclusions:**

Addressing key barriers and enhancing prioritisation of CKD by clinicians and policymakers is critical. Strengthening workforce capacity, awareness, referral systems, and integration into national strategies offers opportunities to improve CKD care.

## Introduction

Chronic kidney disease (CKD) is a growing global public health concern, contributing significantly to morbidity, premature mortality [1–6], and healthcare costs [5–7]. Globally, an estimated 850 million people have CKD [8], accounting for 10% of the world’s population. CKD disproportionately affects low- and middle-income countries (LMICs), where access to preventive and curative care remains limited [11]. In Ethiopia, Emerging hospital-based observational studies indicate that CKD is a common health problem with diabetes, hypertension, and glomerular diseases being the most important causes [12–15].

Previous studies in Ethiopia have described various health system challenges related to the growing burden of chronic diseases, including but not specific to CKD [2,13,16–20]. These include limitations in healthcare infrastructure, access to services, resource constraints, and wider socioeconomic challenges [21,22]. Studies from other countries have identified several barriers and facilitators specific to CKD management, such as healthcare provider knowledge, patient awareness, health system organisation, and policy support [23–25]. However, differences in health system capacity, socioeconomic context, and cultural factors limit the generalisability of these findings to Ethiopia. To our knowledge, there is no existing evidence on the barriers and enabling factors for CKD management in Ethiopia. The objective of this qualitative study was to identify barriers and facilitators in CKD management in Ethiopia from the perspective of healthcare providers and key stakeholders who play a critical role in shaping CKD care.

## Methods

### Study design and setting

A descriptive qualitative study was conducted to explore the perspectives, experiences, and insights of healthcare providers and other stakeholders regarding the barriers and enablers of CKD management. The study’s major emphasis was on healthcare practitioners working at different healthcare levels, primarily in the Amhara region, including tertiary, secondary, and primary levels of healthcare. The Amhara region was purposefully selected for this study as it is one of the most populous regions in Ethiopia with a high burden of CKD [26]. Reporting of this study was guided by the consolidated criteria for reporting qualitative studies (COREQ) checklist [27] (S1 Appendix).

### Participant selection and recruitment

Participants were identified using purposive sampling with maximum variation, supplemented by snowball sampling. Eligible participants were health care providers (general practitioners (GP), nephrologists/internists, and nurses working at the dialysis centre) and non-communicable disease (NCD) program officers and coordinators. We included adults aged 18 years and older, working at least six months in their current role. To confirm a variety of perspectives, diverse recruitment strategies were employed. Initially, we contacted a senior nephrologist (WH) working at tertiary referral centre. We approached participants by sending an invitation letter using personal email and other social media platforms. Diverse participants were approached using a snowball tactic from the primary participants. Potential participants were also invited through department heads with a formal letter. Interested individuals received consent forms and detailed study information by email or preferred channels. The recruitment period was from 15/02/2024 to 20/12/2024. The concept of information power was used to check sample size adequacy, as participants had substantial experience relevant to the study focus [28] (S2 Appendix).

### Data collection

Semi structured interviews were conducted on a purposive sample of 15 participants via Zoom, WhatsApp, or telephone, as appropriate. Each interview lasted approximately 35–54 minutes. The semi-structured interview guide was developed based on a review of documents and guidelines on barriers and facilitators to CKD management (S3 Appendix). Due to the impacts of ongoing armed conflict, especially in the Amhara region, all interviews had completed remotely. The war imposed significant security risks, making travel to certain areas unsafe and limiting the feasibility of in-person interviews. Furthermore, the instability in the region disrupted normal operations in many health facilities, making it difficult for participants to allocate time for face-to-face meetings. Thus, online interviews allowed participants to join from their respective locations without compromising safety [29]. It also enabled the inclusion of diverse perspectives across different levels of healthcare settings, which might not have been possible given the current situation. There were, however, challenges associated with conducting interviews online, including unstable internet connections, limited ability to observe non-verbal cues, and potential distractions in participants’ environments [29,30]. To mitigate these challenges, we maintained flexible scheduling and fostered a supportive interview environment.

The interviews were conducted in Amharic language by DK (PhD candidate), who is trained in qualitative methodologies. The participants had no personal or professional relationship with the interviewer, which allowed participants to express their opinions freely. Interviews were audio recorded and transcribed verbatim by research team members. We also collected demographic and practice information, including participant’s age and number of years in current role, to provide aggregate descriptive information on study participants. Further contextual information is provided in context box below (**Box 1).**

### Reflexivity

The lead researcher (DK), an Ethiopian conducting a PhD at UNSW, reflected on how positionality might shape data collection and interpretation. Being Ethiopian facilitated cultural understanding and rapport with participants, enabling open discussion of sensitive topics, including conflict-related service disruptions. Conducting the study from abroad required careful attention to ensure neutral probing and avoid imposing assumptions. All quotations are presented verbatim to preserve participants’ perspectives, and analysis was conducted collaboratively with the research team to minimise individual bias.

**Box 1: The Context**

*Health System*

Ethiopia, the second-most populous country in Africa, has a three-tiered health system, aiming to provide primary, secondary, and tertiary care. While progress has been made in expanding primary healthcare, access to specialized services remains limited, particularly for chronic conditions like chronic kidney disease (CKD).

*Burden of CKD*

*CKD remains an under-prioritized public health issue, with significant information gap regarding its prevalence, causes, and outcomes* [1,2]. *However, evidence from non-communicable disease (NCD) surveillance programs suggest that the prevalence of key CKD risk factors, such as hypertension and diabetes, is increasing* [3,4].

*Managing CKD in Ethiopia is challenging due to multiple challenges including workforce shortages and lack of dialysis and transplantation services and high out of pocket costs* [1,2]. *Even where treatment is available, the high cost places a significant financial burden on patients, as out-of-pocket expenses remain the primary means of healthcare financing in the absence of universal health insurance*.

*Impact of conflict*

*These gaps in healthcare have been further worsened by ongoing conflicts across various regions of Ethiopia. Armed conflicts have led to the destruction of health infrastructure, displacement of healthcare workers, and disruption in medical supply chains* [9,10]. *The impact of war extends beyond emergency care, severely affecting the management of chronic diseases like CKD. Furthermore, people hesitate to access health services due to security concerns. These challenging conditions, especially the current conflict in the Amhara region, necessitated conducting the interviews online to explore barriers and enablers of CKD management. The war created significant security risks, making travel to certain areas unsafe and limiting the feasibility of in-person interviews. The instability in the region disrupted access to internet and electricity which impacted data collection. We worked closely with interviewees to adjust schedules according to their internet availability and supported them in relocating to areas with more reliable connections, when needed*.

### Conceptual framework

We used the Theoretical Domains Framework (TDF) version 2 to guide the interviews and describe the enablers and barriers derived from the data. TDF synthesises 128 constructs from 33 behaviour and behavioural change theories clustered into 14 domains [31]. Using TDF to explore barriers and enablers of CKD care provides a structured, theory-driven approach to understanding health care providers’ and stakeholders’ behaviours. TDF integrates multiple behaviour change theories into 14 domains, capturing key influences such as knowledge, skills, social influences, and environmental context and resources (S4 Appendix). By mapping data onto TDF domains, the study can link barriers and enablers to evidence-based behaviour change strategies.

### Trustworthiness of the data

To ensure trustworthiness of the data, criteria of credibility, dependability, confirmability, and transferability were applied throughout the research process [32]. Credibility was enhanced through prolonged engagement with the data, iterative coding using NVivo software. Including a diverse participant across disciplines and healthcare levels enhanced transferability. Dependability was ensured through careful coding of transcripts and documentation of coding frameworks in NVivo to support transparency and consistency (S1 Table). To establish confirmability, data were continuously checked and rechecked throughout the study to address any potential biases.

### Data-analysis

Verbatim transcripts of the interviews were reviewed and checked with the audio recordings for accuracy. Transcripts were checked by multiple researchers in the team (DK, RJ, MJ, SK, and WH) who made initial notes and identified potential codes and themes. Transcripts were uploaded to NVivo 14 [33]. We used a deductive strategy guided by the TDF within a reflexive thematic analysis approach to code, interpret, and iteratively refine themes. Transcripts taken from the interview were carefully read and coded independently by two research team members (DK and RJ). An initial coding scheme was created using line-by-line analysis and constant comparison. Transcripts were closely followed to maintain the original meaning of the data. After coding all the transcripts, similar codes were grouped together and rearranged. Transcripts were revisited to ensure all concepts were being captured. Any conflicts in the coding process were resolved through regular discussions until consensus was reached.

### Ethical approval and considerations

Ethical approvals were granted by the University of New South Wales (UNSW) Human Research Ethics Committee (HREC) (iRECS5094) and the Amhara Public Health Institute (RERC: NoH/R/T/T/D/07/59). Written informed consent was obtained electronically before participation. Participants were emailed an information sheet and consent form, which they signed and returned prior to the interview.

## Results

### Demographic characteristics of participants

A total of 15 participants were recruited and included in the analysis: six general practitioners, five nephrologists or internists, one nurse, and one NCD officer and two NCD program coordinators. Among 15 participants, 6 (40%) had been in their current roles for six years or more at the time of interview, and 73% were male. Participants were aged 20–60 years and had between 1 and 15 years of experience in their current roles (S2 Table).

### Relevant TDF domains

**Fig 1:**
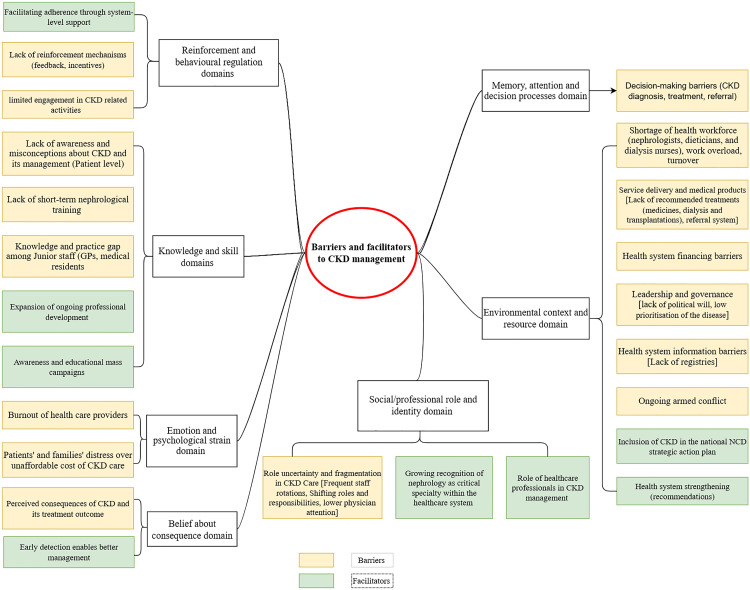
Conceptualising barriers and enablers of CKD management using the Theoretical Domain of Framework.

Thematic analysis identified 8 out of 14 themes aligned with the TDF domains, including environmental context and resources, knowledge and skill, memory, attention and decision processes, beliefs about consequences, emotion and psychological strain, social or professional role and identity, and reinforcement and behavioural regulation (Fig 1).

### Barriers to CKD management

#### Domain 1: Perceived knowledge and skills.

**Lack of awareness** and **misconception about CKD and its management**

Participants described gaps in CKD awareness among patients and healthcare providers, noting how these challenges influence follow-up, adherence, referral practices, and overall disease management. Participants also noted how limited knowledge among primary healthcare providers contributes to delayed recognition and inadequate patient education.

*“Only a small portion of the population living in urban areas are aware of their illness. However, about 80-90% of the population live in rural areas, and based on my experience, their understanding of kidney disease is limited to kidney stones. They do not know the risks for CKD, the signs of CKD, or the treatment options.” (P01, Internist)*

Inadequate clinical assessments prior to treatment were noted by participants, highlighting critical knowledge gaps among healthcare providers. As one nephrologist explained,

*“There was prescription of drugs like iron tablets without checking the haemoglobin status. This points to knowledge gaps, especially among general practitioners and medical residents.” (P02, Nephrologist)*

Participants also highlighted that misconceptions were not limited to the community but were reflected within the health system itself, contributing to the low prioritisation of CKD.

*“CKD is one of the least prioritised diseases, particularly by the government. When a patient is diagnosed with CKD, the general perception is that they will need either dialysis or renal replacement therapy. This thought neglects the importance of other pre-dialysis treatments for CKD, both at the institutional level and within the community. Resources used for CKD treatment, aside from renal transplantation, are often viewed as almost wasteful” (P02, Nephrologist).*

Participants noted that many patients have misconceptions about their treatment needs. As one participant explained.

*“Even those patients on dialysis often believe that dialysis alone is sufficient and do not see the need for ongoing check-ups.” (P09, Nurse).*

### Lack of short term nephrological training

Physicians identified the lack of short-term in-service nephrology training as a critical barrier to CKD management, while other participant emphasised the absence of formal dialysis-specific training opportunities.

*“Even though we are interested in providing better care, we have not received any training specific to dialysis, so we are not fully confident in the service we provide.” (P09, Nurse).*

### Domain 2: Environmental context and resources

Environmental context and resources emerged as a key domain influencing CKD management. Participants identified a range of health system barriers to CKD management, including challenges related to workforce, financing, governance, service delivery, health information systems, and access to essential medicines and technologies.

### Service delivery and medical products

Participants highlighted several service delivery barriers, particularly regarding medication access and medical equipment availability. One key issue raised was the disruption in the supply of medications and essential medical equipment, which impacts the ability to provide consistent care.

*“At the hospital where I currently work, we serve a catchment population of over 10 million people, yet there is not a single dialysis centre. There is no dedicated care unit for CKD, and we do not even have a nephrologist on staff. The government does not prioritise CKD sufficiently, and the number of nephrologists is currently very low.” (P03, Internist).*

### Health information system

Healthcare providers identified challenges in the availability of CKD data, including a lack of registry for CKD.

*“Another important issue is the lack of a data reporting format for CKD that would enable healthcare providers to receive feedback from higher officials. For instance, there are monthly, quarterly, and annual plans for conditions like hypertension and diabetes, and underperformance in these areas requires justification. However, this is not the case for CKD. As a result, healthcare providers tend to overlook CKD” (P10, GP).*

### Health workforce

Health workforce limitations were widely noted, with most participants highlighting critical shortages of trained personnel (nephrologists, dieticians, and dialysis nurses).

*“There is insufficient government attention to CKD care in general, with a critical shortage of nephrologists and other trained healthcare providers. Nephrologists are primarily confined to teaching referral hospitals, while general hospitals increasingly rely on internists to manage cases due to the lack of specialised personnel.” (P06, GP).*

*“Once a patient is diagnosed with CKD, the management goes beyond just medications. It involves other aspects like nutritional therapy, counselling, and financial support, as both medications and lab tests can be very expensive. One major challenge is the lack of a dietitian, so nutritional counselling is provided by non-dietitian personnel.” (P05, Nephrologist)*

### Ongoing armed conflict

Participants noted that ongoing conflict since November 2020 has severely impacted healthcare, leading to medication shortages, supply disruptions, and the turnover of experienced specialists.

*“Over the past three years, the ongoing war has significantly affected NCD care. In some cases, we were unable to manage emergencies because resources, including human power and hospital services, were entirely redirected to support fighters and soldiers. Additionally, many patients on follow-up care discontinued their treatment due to road blockages and widespread fear, further exacerbating the situation” (P12, GP).*

### Leadership and governance

Leadership and governance were identified as key barriers, with participants highlighting the lack of political will, no resource allocation, and low prioritisation of CKD in the national health policy.

*“There are no specific government sectors primarily focused on CKD, which reflects a lack of political commitment. We attempted to include CKD in the major non-communicable disease prevention strategic plan, although it has not been fully implemented. Most of the Ministry of Health programmes are donor-driven” (P13, NCD program coordinator).*

### Domain 3: Emotion and psychological strain

#### Burnout of health care providers.

Participants discussed the significant challenges of managing CKD, emphasising the psychological strain it places on healthcare professionals. Furthermore, due to the shortage in the health workforce, healthcare providers often raised the issue of high workload and feeling burnt out. One explained,

*“Most CKD patients cannot afford the high cost of transplantation, and many only receive treatment through fundraising efforts organised by social workers. For us physicians, telling a patient they have CKD often feels like “delivering a death sentence,” as treatments like dialysis and transplants are out of reach for most patients due to their prohibitive costs.” (P05, Nephrologist).*

#### Patients and families experiencing distress over cost of CKD care.

Participants shared heartbreaking experiences of managing CKD in resource-limited settings, where financial barriers and conflict severely limit access to care.

*“A young adult female with advanced kidney disease, living some distance from the facility (compounded by the challenges of transportation due to the ongoing war), presented in clinical encounter, she was diagnosed for CKD and discussed potential treatment options like vascular access and kidney transplantation, stressing the need for referral. Subsequently, she and her family were crying, because they do not afford the costs. They live 30-40 km away, compounded by the challenges of transportation due to the ongoing war. Imagine the hardship for this young woman. Regrettably, she will not be able to sustain dialysis care either. This scenario is emblematic of the difficulty faced by many CKD patients in Ethiopia. Despite informing them about available treatment options post-referral, financial constraints often prevent them from accessing care” (P01, Internist).*

#### Domain 4: Social/professional role and identity.

Participants reported unclear roles and lack of ownership for CKD care, with no strong professional leadership to drive advocacy. As one participant explained:

*“There is no strong professional organization in nephrology capable of pressuring the government to prioritize CKD management.” (P13, NCD program coordinator).* “*Physicians often give less attention to non-communicable diseases compared to communicable diseases. This reflects limited prioritization within professional roles and contributes to low prevention effort.” (P12, GP)*

### Domains 5 and 6: Reinforcement and behavioural regulation

Participants noted limited engagement in CKD activities due to a lack of structured follow-up, incentives, and accountability mechanisms.

*“There are monthly, quarterly, and annual plans for conditions like hypertension and diabetes... however, this is not the case for CKD. As a result, healthcare providers tend to overlook CKD.” (P10, GP).* “*There is no sponsorship opportunity for this renal program, as a result, due to financial constraints, there is no interest in joining the program.” (P01, Internist)*

### Domain 7: Memory, attention and decision processes

Participants highlighted challenges related to the cognitive demands of CKD diagnosis and management. Difficulties in retaining and applying clinical guidelines, recognising non-specific symptoms, and making timely decisions about disease staging and treatment planning were frequently noted.

*“Another issue is improper staging of CKD using the Kidney Disease: Improving Global Outcomes (KDIGO) guidelines, which involve eGFR and albuminuria. There’s also a lack of early planning for kidney replacement therapy, which is a critical problem for CKD patients” (P03, Internist).*

### Domain 8: Belief about consequences

Healthcare providers highlighted several misconceptions and concerns that influence how CKD is perceived and managed. They noted that many patients fear dialysis will severely impact their quality of life, leading some to refuse treatment altogether. Others believe CKD is an untreatable condition, turning to traditional medicine instead. Additionally, societal and institutional perspectives often focus primarily on dialysis and transplantation, overlooking other pre-dialysis treatments.

*“Besides the financial burden, some patients are reluctant to receive dialysis. They feel that lifelong dialysis will impact their family, social life, and economic well-being. In my experience, most patients become unable to continue their regular activities after starting dialysis — not because of the treatment itself, but due to the emotional, financial, and social burdens associated with it.” (P09, Nurse)*

### Facilitators/enablers to CKD management

Enablers to the management of CKD were categorised into five domains of the TDF domains.

### Knowledge and skill of health care providers and patients

Participants identified various facilitators related to knowledge and skills that support CKD management. These include increased access to specialised training (relative to the past), and use of KDIGO guideline by nephrologists. Additionally, they highlighted the importance of ongoing professional development, policy-driven awareness campaigns, and collaborative initiatives to improve CKD care.

*“In collaboration with the Ministry of Health, it is good to work on advocacy by posting leaflets, providing education for the community about CKD screening and symptoms, and through different channels like social media and mainstream media” (P05, Nephrologist). “While some programs exist on mainstream media, they are insufficient to meet the need.” (P11, GP)*

### Environmental context and resources

Participants identified several facilitators under environmental context and resource domain. The inclusion of CKD in the national NCD strategic action plan and ongoing advocacy efforts (leadership and governance) and the relative growing number of nephrologists (though still limited) was highlighted.

*“The presence of a strategic plan for CKD, along with a revised health policy that encourages decentralised services, which is crucial for early screening and management of CKD.” (P13, national-level NCD program lead). “Screening and treatment have also started at the lower levels of the healthcare system (health centres), which is important for preventing CKD. This ensures that patients can access treatment closer to their home.” (P10, GP)*

*“One opportunity I recall is a new initiative called the Public-Private Partnership Dialysis Centre, which provided dialysis services for both AKI and CKD patients. This initiative improved the availability of consumable supplies and allowed a greater number of patients to access the services.” (P04, Nephrologist)*

### Social/professional role and identity

Participants highlighted the increasing recognition of nephrology as a vital specialty within the healthcare system, while also pointing to structural barriers in professional pathways.

*“A system should be introduced to allow general practitioners to join nephrology programmes directly, rather than first specialising as internists. The current pathway, requiring GPs to become internists before subspecialising in nephrology, is time-consuming” (P10, GP)*

### Beliefs about consequences

Participants underscored the value of expanding laboratory services, particularly creatinine testing in primary care, and highlighted the benefits of early detection and staging for improved CKD management.

*“Early detection and staging based on KDIGO guidelines lead to better management.” (P04, Nephrologist)*

### Behavioural regulation and reinforcement

Participants emphasised that improving outcomes requires deliberate efforts to promote adherence through system-level interventions*.*

*“Promoting adherence to treatment through advocacy, education, and policy changes is critical to improving outcomes” (P04, Nephrologist)*

The summary of themes with illustrative quotes for facilitators and barriers to CKD management is presented in **Table 1** and **Table 2** respectively.

**Table 1:** Summary of themes and illustrative quotes for barriers to CKD management.

TDF Domains	Sub-theme (Barriers)	Illustrative quotes
**Knowledge and skill**	Lack of awareness, misconception, about CKD and its management (Patient level)	*“Only a small portion of the population living in urban areas are aware of their illness. However, about 80-90% of the population live in rural areas, and based on my experience, their understanding of kidney disease is limited to kidney stones. They do not know the risks for CKD, the signs of CKD, or the treatment options. There is no appropriate educational channel for the community about this. In addition, we do not have uniform educational and registry guidelines for the disease.” (P01, Internist)*
		*“Instead of attending the formal management plan, some patients turn to traditional healers and cultural practices for their treatment. Since CKD is a chronic condition requiring long-term follow-up and treatment, patients may not see immediate improvement. As a result, they sometimes choose to cancel their appointments and seek alternatives like holy water rituals with their religious leaders.” (P12, GP)*
		*“Despite our extensive efforts to provide mass education through media campaigns and workshops on NCD prevention, community awareness remains limited. Many individuals are hesitant to undergo NCD screening, accept a diagnosis, or commit to a management plan.” (P15, Regional NCD officer)*
		*“There is a common misconception in the community that starting medication, particularly injections, will worsen the condition. They believe it is caused by the devil or possibly God’s punishment, and therefore, medicine cannot cure it. This makes the management plan very challenging.” (P14, GP)*
	Lack of short term nephrological training	*“I did not take any CKD related training. However, I have attended training specifically on type 1 diabetes. The training covered screening and early detection of diabetes, preventing complications, and providing proper treatment for those already diagnosed.” (P06, GP)*
		*“Even though we are interested in providing better care, we have not received any training specific to dialysis, so we are not fully confident in the service we provide.” (P09, Nurse)*
	Knowledge and practice gap among GPs and medical residents	*“There are also knowledge gaps among healthcare providers. Many general practitioners fail to consider CKD as a differential diagnosis, largely due to a lack of training on the condition.” (P10, GP).*
		*“We try our best to handle all referred cases that come to our hospital. However, there is knowledge gap among general practitioners (GPs) in peripheral hospitals regarding risk stratification and the appropriate timing for referrals. Many GPs tend not to refer CKD cases until dialysis is required.” (P05, Nephrologist)*
		*“There was level of awareness at lease at the higher technical and policy level and CKD is already recognised as important public health problem by the federal authority.” (P08, NCD Program coordinator, WHO)*
**Environmental context and resources**	Shortage of health workforce [(nephrologists, dietician, and dialysis nurse), work overload, turnover]	*“There is insufficient government attention to CKD care in general, with a significant shortage of nephrologists and other trained healthcare providers. Nephrologists are primarily confined to teaching referral hospitals, while general hospitals increasingly rely on internists to manage cases due to the lack of specialized personnel.” (P06, GP)*
		*“We only have 12 nurses, none of whom are trained specifically in dialysis or CKD care. This means dialysis services are provided by untrained staff, relying solely on their experience. There is no nurse assigned permanently to the dialysis centre.” (P09, Nurse)*
		*“Lack of trained staff (Nephrologist), which is budget intensive is the main challenge for CKD care. The other unique challenges we face in this year is the turnover of senior health care providers mostly with specialities. This will be continued problem related to conflict.” (P15, Regional NCD officer)*
		*“From the physician’s perspective, the high workload and case overload hinder the delivery of appropriate care to patients.” (P12, GP)*
	Service delivery and medical products [Lack of recommended treatments (medicines, dialysis and transplantations), referral system]	*“The referral practice is very poor. There is a notable deficiency in proactive referral by general practitioners (GPs) and internists, who often decide to manage CKD cases independently rather than referring them to specialists like nephrologist.” (P02, Nephrologist)*
		*“We serve a catchment population of over 10 million people, yet there is not a single dialysis centre. There is no dedicated care unit for CKD.” (P03, Internist)*
		*“Government hospitals do not provide dialysis services for CKD patients. As a result, many patients are unable to receive this critical treatment.” (P04, Nephrologist)*
		*“The availability and affordability issues of essential medications are significant challenges for CKD care. There are effective medications like SGLT2, but they are beyond the financial reach of our patients.” (P04, Nephrologist)*
		*“Most CKD patients cannot afford the high cost of transplantation, and many only receive treatment through fundraising efforts organized by social workers. For us physicians, telling a patient they have CKD often feels like “delivering a death sentence”, as treatments like dialysis and transplants are out of reach for most patients due to their prohibitive costs.” (P05, Nephrologist).*
		*“We are often limited by machine breakdowns, meaning some patients must go home without receiving dialysis until the machines are repaired. We are also facing a shortage of consumable materials, like dialysers.” (P09, Nurse)*
		*“There are shortage of laboratory tools, medications and other equipment’s which are essential for CKD management. There is no dialysis centre in our area, forcing patients to travel 300–400 kilometres for dialysis services.” (P11, GP)*
	Health system financing barriers	*“The so-called community health insurance in Ethiopia, which mostly applicable in the rural community, but this service did not cover the expenses of dialysis since it is very expensive.” (P01, Internist)*
		*“Compared to other diseases, the cost of managing CKD in our country is significantly higher, making it a major challenge.” (P02, Nephrologist).*
		*“I attempted to enrol in a nephrology program at an institution two years ago, but financial constraints forced me to discontinue it. However, I firmly believe that an appropriate number of professionals should be trained and deployed to address the needs of our large population.” (P01, Internist)*
		*“In Ethiopia, the total annual budget for NCDs is less than $2 million. This inadequate funding means that most NCDs, including CKD, receive little to no financial support. The focus in Ethiopia remains heavily skewed toward infectious diseases, and even among global actors, the emphasis is largely on hypertension and diabetes, with little consideration for CKD.” (P08, NCD Program coordinator, WHO)*
		*“Most of the Ministry of Health programs are donor-driven, and within the NCD sector, the focus has shifted towards cancer due to the availability of global funding (From the government’s NCD budget allocation, 50-60% of the funds are directed towards cancer in addition to doners). If funds were available for CKD, there would be no reason not to work on it.” (P13, NCD program coordinator).*
	Leadership and governance	*“There are no specific government sectors primarily focused on CKD, which reflects a lack of political commitment. Additionally, there is no strong professional organization in nephrology capable of pressuring the government to prioritize CKD management.” (P13, NCD program coordinator)*
		*“I believe that when policies are being set, there may not be sufficient involvement from CKD experts. While it’s well-known that CKD is one of the leading causes of death, it does not receive the attention it deserves. Most financial resources tend to be allocated to infectious diseases rather than NCD including CKD.” (P03, Internist)* *The government may not fully understand the burden of the disease.” (P05, Nephrologist)*
		*“In Ethiopia, most of our policies, strategies, and public health programs are shaped by global directives. While developed countries have their own data, policies, and strategies to address CKD, countries like Ethiopia, which lack such resources, face challenges in effectively addressing the issue.” (P08, NCD Program coordinator, WHO)*
	Health system information barriers	*“To my knowledge, there is no national data on CKD.” (P05, Nephrologist)*
		*“It is also important to train staff on proper data recording for CKD cases.” (P07, GP)*
		*“Another important issue is the lack of a data reporting format for CKD that would enable healthcare providers to receive feedback from higher officials. For instance, there are monthly, quarterly, and annual plans for conditions like hypertension and diabetes, and underperformance in these areas requires justification. However, this is not the case for CKD. As a result, healthcare providers tend to overlook CKD.” (P10, GP)*
	Ongoing armed conflict	*“The ongoing war in the area greatly impacts transportation. It’s very difficult for patients to find transport, and even if they manage to, they often face delays due to road blockages. This can significantly hinder their ability to reach the clinic in a timely manner after referral.” (P01, Internist)*
		*“There is no structured approach to managing NCDs in Ethiopia during crises. Many deaths from conditions like hypertension and diabetes occur due to a lack of essential medications, such as insulin, often caused by factors like road blockages and other disruptions.” (P08, NCD Program coordinator, WHO)*
		*“Over the past three years, the ongoing war has significantly affected NCD care. In some cases, we were unable to manage emergencies because resources, including human power and hospital services, were entirely redirected to support fighters and soldiers.” (P12, GP)*
**Emotion and psychological strain**	Burnout of health care providers	*“When a patient cannot afford the costs of referral care, it presents a profound moral dilemma for healthcare providers. We do our best to offer all possible care within our facility.” (P01, Internist),*
		*“Many CKD patients arrived at hospital with critical condition, with severe symptoms like uraemia and vomiting blood, and we are left helpless due to the lack of equipment. Managing CKD here is extremely challenging and takes a psychological stress on us as healthcare professionals.” (P09, Nurse)*
	Patients and families experience distress over unaffordable CKD care	*“Another challenge comes from the patients and their families, as well as the broader community. When a family member is diagnosed with CKD, both the patient and their family often lose hope. Many patients believe there is no treatment available for CKD, so they choose to go home rather than seeking further medical care. Families are also reluctant to spend money on treatment, as they assume the patient will not survive for long.” (P10, GP)*
**Social or professional role and identity**	Role uncertainty and fragmentation in CKD Care [Frequent staff rotations, Shifting roles and responsibilities, lower physician attention]	*“There is no strong professional organization in nephrology capable of pressuring the government to prioritise CKD management.” (P13, National-level NCD program lead)*
		*“Physicians often give less attention to non-communicable diseases compared to communicable diseases. This reflects limited prioritization within professional roles and contributes to low prevention effort.” (P12, GP)*
		*“There is no nurse assigned permanently to the dialysis centre; staff rotate in from other departments. Nurses should also be permanently assigned to the dialysis centre. As I mentioned earlier, when nurses are assigned to dialysis, they receive no formal training—they just work based on shared experience. Unfortunately, once they gain experience, they are reassigned to other wards, and new, inexperienced staff are brought in.” (P09, Nurse).*
		*“Community-based activities, including screening, are not part of our responsibilities as a referral hospital. This is due to the country’s health policy, which assigns such tasks to health centres and primary hospitals. Referral hospitals focus more on treatment and curative services.” (P11, GP)*
		*“My primary role involves patient evaluation, clerking, initiating management, and conducting follow-ups. From an NCD perspective, I provide care for conditions such as hypertension, diabetes, CKD, and other cardiac cases.” (P12, GP)*
**Reinforcement and behavioural regulation**	Lack of reinforcement mechanisms (feedback, incentives)	*“There is no sponsorship opportunity for this renal program, as a result, due to financial constraints, no interest to join the program.” (P01, Internist)*
		*“To be honest, we do not have any organised or formal community screening programs for CKD. Occasionally, there are voluntary initiatives organized by the health bureau, but these are usually limited to screening for hypertension or diabetes.” (P12, GP)*
**Memory, attention and decision processes**	Decision making barriers (CKD diagnosis, treatment, referral)	*“Another issue is improper staging of CKD using the KDIGO guidelines, which involve eGFR and albuminuria. There’s also a lack of early planning for renal replacement therapy, which is a critical problem for CKD patients.” (P03, Internist).*
		*“In practice, diagnosing CKD is often a dilemma. As I mentioned earlier, some patients don not present with acute symptoms, making diagnosis difficult. It often depends on the patient’s follow-up history.” (P04, Nephrologist).“If the GFR drops below 15% and the patient develops end-stage renal disease, we refer them for renal replacement therapy.” (P06, GP)*
**Belief about consequence**	Perceived consequences of CKD and its treatment	*“Besides the financial burden, some patients are reluctant to receive dialysis. They feel that lifelong dialysis will impact their family, social life, and economic well-being.” (P09, Nurse)*
		*“Some patients believe that CKD is an untreatable condition, so they turn to traditional medicines instead.” (P03, Internist)*
		*“They (CKD patients) feel that lifelong dialysis will impact their family, social life, and economic well-being. So, they prefer not to start dialysis at all., They also question, “How long will I have to continue with dialysis? What’s the end point?” (P09, Nurse)*

NCD: Non-Communicable Disease; CKD: Chronic Kidney Disease; WHO: World Health Organisation, GP: General Practitioner.

**Table 2:** Summary of themes and illustrative quotes for facilitators to CKD management.

TDF domains	Sub-themes	Illustrative quates
**Knowledge and skill**	Awareness and Education Campaigns	*“Growing use of KDIGO guidelines for CKD diagnosis and staging.” (P02, Internist)*
		*“We provide health education more frequently, and treatment-related challenges, such as insulin shortages, have been resolved.” (P06, GP)*
		*“First, we mostly adhere to the definition of CKD using KDIGO guidelines. We assess proteinuria, imaging, and other renal function tests.” (P03, Internist)*
		*“Providing CKD training for general practitioners after graduation, like HIV training, would be beneficial.” (P03, Internist)*
		*“In collaboration with the Ministry of Health, it is good to work on advocacy by posting leaflets, providing education for the community about CKD screening, symptoms, and through different channels like social media and mainstream media” (P05, Nephrologist)*
		*‘More on, the healthcare workers should work with the government and create awareness, this is very important.” (P05, Nephrologist)*
		*“As a policy initiative, incorporating podcasts into health system advocacy efforts is essential for raising awareness among the broader population. While some programs exist on mainstream media, they are insufficient to meet the need.” (P11, GP)*
		*“Senior staff have started media campaigns to raise awareness about the disease, which is promising.” (P07, GP)*
	Training and Knowledge Enhancement	*‘I have attended training specifically on type 1 diabetes. The training covered screening and early detection of diabetes, preventing complications, and providing proper treatment for those already diagnosed.” (P06, GP)*
		*“There is a relatively good level of awareness about the disease among healthcare providers.” (P11, GP)*
**Environmental Context and Resources (Availability of infrastructure, financial and policy support)**	Health system strengthening (Leadership and governance, health workforce, health system financing, medical products, and technologies)	*“CKD should be prioritised just like HIV and TB, which receive more funding. The government does not prioritize CKD sufficiently, and policies do not involve CKD experts in the planning process.” (P03, Internist)*
		*“We need more advocacy to shift the government’s focus on CKD management, especially in conflict-affected regions.” (P04, Nephrologist)*
		*“We have begun working on establishing a nephrology association in Ethiopia. This will facilitate better communication and help shift the focus of the government and other stakeholders toward addressing CKD.” (P05, Nephrologist)*
		*“CKD has been included in our recent national guidelines. CKD is already recognized as an important public health problem by the federal authority (MoH).” (P08, NCD Program coordinator, WHO)*
		*“The presence of a strategic plan for CKD, along with a revised health policy that encourages decentralized services, which is crucial for early screening and management of CKD.” (P13, National-level NCD program lead lead)*
		*‘Dialysis care was previously provided by general practitioners after a brief three-month training, but now we are seeing more specialized training opportunities.” (P01, Internist)*
		*“Compared to the past, the number of senior staff, like nephrologists, is increasing.” (P07, Nephrologist*
		*“We are working closely with key hospitals, which serve as the main referral centres for CKD.” (P08, NCD program coordinator, WHO)*
		*“The government should allocate a budget to support CKD-related training for both healthcare providers and patients.” (P10, GP)*
		*“Regarding healthcare professionals, nephrologists are now available in some selected hospitals, which is an improvement from before.” (P12, GP)*
		*“Comparatively, there has been some improvement in the number of nephrologists and internists compared to the past. Additionally, the government and health sectors are looking to support and collaborate with external partners.” (P14, GP)*
		*“Dialysis solutions should be fully covered or subsidized by the government since most patients cannot afford them.” (P02, Nephrologist)*
		*“There’s been some progress in diagnostic access for CKD, such as tests for proteinuria and erythropoietin at the hospital level.” (P03, Internist)*
		*“One opportunity I recall is a new initiative called the Public-Private Partnership dialysis Centre, which provided dialysis services for both AKI and CKD patients. This initiative improved the availability of consumable supplies and allowed a greater number of patients to access the services.” (P04, Nephrologist)*
		*“It would be beneficial to have a dedicated dialysis ward with its own OPD, emergency services, laboratory, and admission facilities.” (P09, Nurse)*
		*“Screening and treatment have also started at the lower levels of the healthcare system (health centres), which is important for preventing CKD. This ensures that patients can access treatment closer to their homes.” (P10, GP)*
		*“Dedicated nephrology units should be established, including separate outpatient departments, follow-up units, and dialysis units.” (P10, GP)*
		*“Referral hospitals should establish dedicated nephrology units to adequately serve their catchment populations.” (P11, GP)*
		*“The expansion of health facilities has shown promising progress compared to the past.” (P12, GP)*
		*“Consumables like dialysis solutions should be fully covered or subsidised.” (P05, Nephrologist)*
		*.” Improving access to certain medications for CKD patients, like GLP-1 inhibitors, presents another opportunity for better management.” (P03, Internist)*
**Social/Professional Role and Identity**	Growing recognition of nephrology as a critical specialty within the healthcare system	*“As there was no nephrologist in our institution before, having a nephrologist and patients being referred to a nephrologist and getting treatment is a good opportunity.” (P02, Nephrologist)*
	Role of healthcare professionals in CKD management	*“Healthcare providers should work on prevention... advocate for CKD awareness.” (P04, Nephrologist)*
		*“A system should be introduced to allow general practitioners to join nephrology programs directly, rather than first specializing as internists. The current pathway, requiring GPs to become internists before subspecializing in nephrology, is time-consuming.” (P10, GP)*
		*“Nephrologists need to apply pressure by establishing professional associations to influence policy.” (P04, Nephrologist)*
**Beliefs About Consequences**	Facilitating adherence through system-level support	*“Expanding laboratory services, especially for creatinine testing in primary care, is crucial.” (P03, Internist)*
		*“Early detection and staging based on KDIGO guidelines lead to better management.” (P04, Nephrologist)*
**Behavioural regulation and enforcement**	Early detection enables better management	*“Promoting adherence to treatment through advocacy, education, and policy changes is critical to improving outcomes.” (P04, Nephrologist)*

KDIGO: Kidney Disease: Improving Global Outcomes; CKD: Chronic Kidney Disease; NCD: Non-Communicable Disease, GP: General Practitioner; WHO: World Health Organisation.

## Discussion

This descriptive qualitative study explored potential barriers and facilitators that affect CKD care practices across the healthcare system. Identified barriers include patient misconceptions, low patient and healthcare provider awareness, shortage of nephrology workforce, knowledge gaps, limited resources, high out-of-pocket costs, lack of CKD registries, weak referral systems, inconsistent access to medicines and diagnostics, lack of structured training, and conflict-related disruptions. Identified facilitators includes the use of KDIGO guideline by senior staff, the inclusion of CKD into national major NCD prevention strategies, and increased use of media for mass health education. These findings identify areas where targeted interventions could improve the quality of CKD care.

### Health system-related barriers

The lack of service delivery for the prevention and control of CKD at the primary health care level, which is often the first point of contact for communities, is similar to other low-resource settings [34,35]. Participants described that healthcare providers at this level, including general practitioners, frequently lack adequate knowledge and training in CKD screening, diagnosis, and management. This is compounded by a lack of clinical guidelines and limited opportunities for professional development. In contrast, nephrologists and internists reported using KDIGO guidelines, which they found beneficial for guiding evidence-based and consistent care [25]. Similar to our study findings, research from other LMICs have also described gaps in provider knowledge and challenges in delivering CKD care at the primary level [23,36,37]. Participants in this study underscored the importance of targeted sustainable educational initiatives, including those delivered during medical residency and through workplace-based training, as critical strategies to strengthen CKD management competencies at the primary care level.

Another recurrent barrier was the critical shortage of specialised workforce for kidney care, including nephrologists, dietitians, and dialysis nurses. For instance, respondents noted that there were only two nephrologists available in the entire Amhara region. This aligns with national data showing that Ethiopia has an extremely limited nephrology workforce, with a ratio of 0.26 nephrologists per million population [15]. Added upon this shortage, most of these nephrologists are based in major cities, leaving many regions without nephrologists. Our findings are further supported by the Global Kidney Health Atlas report, which highlights stark disparities in nephrology workforces globally, with 80-fold more nephrologists in high- vs. low-income countries [35,38]. In addition, participants noted that most nurses staffing dialysis units lack formal training; they often receive only informal, hands-on training and tend to move onto other rotations rather than specialising in dialysis care. This lack of structured training contributes to substandard care, as similarly noted in global perspective on dialysis services in Ethiopia [15].

Participants in our study described dialysis care as largely inaccessible and unaffordable. This reflects the severe shortage of dialysis infrastructure in Ethiopia, where a 2021 survey reported only 35 haemodialysis units nationwide, two-thirds of which are located in Addis Ababa, home to just 4.3% of the population [15]. Many regions lack any dialysis services, and where they do exist, patients often face high out-of-pocket costs. Reported charges for a dialysis session in private facilities typically range from approximately USD 42–55 (prior to currency devaluation) [15,39]. Taken together, these findings underscore that dialysis costs are prohibitively high relative to average household income, leaving most patients reliant on private providers and facing catastrophic out-of-pocket expenditure. These barriers mirror broader challenges reported across sub-Saharan Africa, where dialysis infrastructure is scarce, utilities like water and electricity are unreliable, and financial and geographic barriers remain high [40]. Studies from the broader African context also emphasise the difficulty of expanding dialysis within limited healthcare budgets and competing health priorities [41,42].

The participant noted that although CKD is included in Ethiopia’s NCD strategic action plan [43], its implementation is slow and poorly coordinated. Building on this, participants identified systemic barriers that continue to hinder progress, including limited political commitment, inadequate financing, and weak health information systems, marked by poor documentation, limited digital infrastructure, lack of CKD-specific registries, and scarcity of locally relevant data. These findings align with challenges documented in other LMICs, where NCDs like CKD often remain under-prioritised in national health agendas [38,44], and poor health information systems [13,36,37]. This may contribute to late diagnoses, fragmented service delivery, and an overall lack of structured programmes to address the growing burden of kidney disease [38].

### Patient level barriers

Limited awareness and poor understanding of CKD, misconceptions about the disease, and inadequate adherence to treatment recommendations were the main patient level barriers identified. As clearly highlighted by the respondents, CKD is often perceived as a punishment for past actions, often attributing the disease to divine retribution. This leads to poor health seeking behaviour for the patients. Furthermore, previous studies documented that patients often prefer traditional medicine, due to cultural beliefs, mistrust of conventional treatments, or financial problems [45,46]. Health literacy, including awareness about disease progression and risk factors, is a central part of contemporary medicine [47,48]. Our findings highlight that targeted strategies to improve awareness and understanding of CKD may support better management and treatment outcomes.

### Contextual challenges

Study participants described how contextual issues, particularly the ongoing armed conflict in Ethiopia, have severely impacted the provision of CKD care. As described, the conflict had disturbed routine health services, especially in the affected regions, through the destruction of health facilities, displacement of healthcare workers, and insecurity that limits access to both patients and providers. Prior studies indicated that loss of infrastructure and staff displacement disrupt the continuity of care [49–51]. As some participants shared, patients were left without access to regular follow-up, medication, even basic health information. This was seen in chronic conditions like CKD, which require ongoing monitoring and consistent treatment [52,53]. Patients in these settings were faced difficulty to maintain regular contact with healthcare providers which place them increased risk of disease progression [54,55]. Most importantly, there is no structured approach to managing NCDs in Ethiopia during crises. These contextual challenges are consistent with previous findings highlighting how conflict settings weaken healthcare systems to handle chronic conditions [52].

### Participants’ insights on strengthening CKD management

Study participants underlined several actionable suggestions to improve CKD care in Ethiopia. Inclusion of CKD in the national NCD prevention strategies was recommended as a major step toward prevention and long-term care. The participant advocated for dedicated nephrology units and decentralised CKD services to improve access across healthcare levels. Participants also called for government subsidies for dialysis service and improved access to essential medications. However, these efforts need strong political will, continual investment in the health workforce, and robust health information systems to support implementation and track the disease progress.

### Strengths and limitations of the study

Including participants from diverse healthcare levels and professional backgrounds provided a broad perspective that enriched the findings. However, the study also has limitations. The small sample size, impacted by the ongoing armed conflict, may limit the generalisability of the findings. Additionally, the use of online interviews, though necessary due to the conflict, may have introduced selection bias related to internet access, and differences in communication dynamics compared to in-person interviews. Perspectives from dietitians, pharmacists, patients and other health cadres were not captured and should be explored in future research. Similarly, the study did not capture perspectives from all regions of Ethiopia, which may limit the breadth of regional representation; this remains an important area for future research to strengthen the generalisability of findings across the country. Finally, member checking was not conducted, which we acknowledge as a limitation, though trustworthiness was ensured through established qualitative rigor criteria.

## Conclusions

Several barriers to effective CKD management require critical attention. Greater prioritisation of CKD by clinicians and policymakers is essential. Multipronged strategies should strengthen workforce capacity, improve patient and provider awareness, and enhance referral pathways and access to care. Expanding structured training and integrating CKD into national strategies may offer important opportunities for improvement.

## Supporting information

S1 Table NVivo node-by-node frequency table showing the number of references per participant (P01–P15) for included TDF domains. (DOCX)

S2 Table Participants demographic and practice characteristics. (DOCX)

S1 Appendix Consolidated criteria for reporting qualitative studies (COREQ) checklist. (DOCX)

S2 Appendix Justification of sample size using Malterud’s information power framework. (DOCX)

S3 Appendix Interview guide. (DOCX)

S4 Appendix Description of Theoretical Domains Framework. (DOCX)

## Abbreviations

CKD: (chronic kidney disease)
GP: (general practitioner)
KDIGO: (Kidney Disease: Improving Global Outcomes)
LMIC: (low-and middle-income countries)
NCD: (non-communicable disease)
TDF: (Theoretical Domains Framework)
