# Supplementary material for: Stakeholders’ perspectives on barriers and enablers of chronic kidney disease care in Ethiopia: A qualitative study
Source: PLoS One. 2025 Nov 13;20(11):e0336781. doi: 10.1371/journal.pone.0336781 (PMC12614622; doi:10.1371/journal.pone.0336781)
Supplement: S1 Table — (DOCX) [file pone.0336781.s001.docx]

**S1 Table** : NVivo node-by-node frequency table showing the number of references per participant (P01–P15) for included TDF domain

| **Node / Code (TDF Domain)** | **Study participants [Code : P01-P15]** | | | | | | | | | | | | | | | **Total Frequency** |
| --- | --- | --- | --- | --- | --- | --- | --- | --- | --- | --- | --- | --- | --- | --- | --- | --- |
|  | **P01** | **P02** | **P03** | **P04** | **P05** | **P06** | **P07** | **P08** | **P09** | **P10** | **P11** | **P12** | **P13** | **P14** | **P15** |  |
| Knowledge and skill | 6 | 4 | 10 | 0 | 4 | 3 | 9 | 3 | 4 | 3 | 6 | 3 | 0 | 3 | 2 | 62 |
| Environmental context and resources | 12 | 10 | 4 | 2 | 9 | 4 | 9 | 10 | 2 | 4 | 2 | 1 | 5 | 4 | 7 | 85 |
| Emotion and psychological strain | 3 | 4 | 1 | 2 | 1 | 1 | 4 | 0 | 3 | 1 | 2 | 1 | 0 | 2 | 0 | 25 |
| Social or professional role and identity | 1 | 2 | 0 | 2 | 0 | 2 | 1 | 1 | 2 | 1 | 3 | 2 | 0 | 0 | 0 | 17 |
| Reinforcement and behavioral regulation | 2 | 3 | 1 | 1 | 0 | 1 | 2 | 1 | 0 | 2 | 0 | 1 | 0 | 1 | 1 | 16 |
| Memory, attention and decision processes | 0 | 1 | 2 | 2 | 0 | 2 | 1 | 0 | 0 | 0 | 0 | 1 | 0 | 0 | 0 | 9 |
| Belief about consequence | 0 | 1 | 2 | 1 | 0 | 0 | 1 | 0 | 2 | 1 | 0 | 0 | 0 | 1 | 0 | 9 |

*Numbers represent the count of coded references (text segments) from each participant’s transcript that were assigned to the respective TDF domain; totals indicate the sum of all coded references across participants.* *For example, the value ‘12’ under Participant 01 in the ‘Environmental context and resources’ domain indicates that 12 text segments from this participant were coded to this domain, while the total ‘85’ represents the sum of all coded segments across participants*
