# Supplementary material for: Stakeholders’ perspectives on barriers and enablers of chronic kidney disease care in Ethiopia: A qualitative study
Source: PLoS One. 2025 Nov 13;20(11):e0336781. doi: 10.1371/journal.pone.0336781 (PMC12614622; doi:10.1371/journal.pone.0336781)
Supplement: S2 Table — (DOCX) [file pone.0336781.s002.docx]

**S2 Table:** Participants demographic and practice characteristics

| **Code** | **Age range** | **Gender** | **role** | **Working Institution** | **Duration in the current role (years)** |
| --- | --- | --- | --- | --- | --- |
| P01 | 30-40 | Male | Specialists | Tertiary level healthcare | 1-5 |
| P02 | 30-40 | Male | Specialists | Tertiary level healthcare | 1-5 |
| P03 | 30-40 | Male | Specialists | Tertiary level healthcare | 6-10 |
| P04 | 30-40 | Female | Specialists | Tertiary level healthcare | 1-5 |
| P05 | 30-40 | Female | Specialists | Tertiary level healthcare | 6-10 |
| P06 | 30-40 | Male | GP | Secondary level healthcare | 6-10 |
| P07 | 20-29 | Female | GP | Tertiary level healthcare | 1-5 |
| P08 | 40-50 | Male | NCD program coordinator | Health administrator (WHO) | 11-15 |
| P09 | 30-40 | Female | Dialysis nurse | Tertiary level healthcare | 6-10 |
| P10 | 30-40 | Male | GP | Secondary level healthcare | 1-5 |
| P11 | 30-40 | Male | GP | Primary level healthcare | 1-5 |
| P12 | 30-40 | Male | GP | Primary level healthcare | 1-5 |
| P13 | 51-60 | Male | NCDs program coordinator | Health administrator (MoH) | 11-15 |
| P14 | 20-29 | Male | GP | Primary level healthcare | 1-5 |
| P15 | 30-40 | Male | Regional NCDs officer | Health administrator (NCD prevention and control officer) | 1-5 |

GP: General practitioner; NCD: Non-Communicable Disease; WHO: World Health Organization; MoH: Ministry of Health
