# Supplementary material for: Stakeholders’ perspectives on barriers and enablers of chronic kidney disease care in Ethiopia: A qualitative study
Source: PLoS One. 2025 Nov 13;20(11):e0336781. doi: 10.1371/journal.pone.0336781 (PMC12614622; doi:10.1371/journal.pone.0336781)
Supplement: S1 Appendix — (DOCX) [file pone.0336781.s003.docx]

**S1 Appendix:**  Consolidated criteria for reporting qualitative studies (COREQ) checklist

| **No.** | **Item** | **Description** | **Section #** |
| --- | --- | --- | --- |
| **Domain 1: Research team and reflexivity** | | | |
| Personal characteristics | | | |
| *1.* | Interviewer/facilitator | Which author/s conducted the interview or  focus group? | 6 |
| *2.* | Credentials | What were the researcher's credentials? *E.g.*  *PhD, MD* | 6 |
| *3.* | Occupation | What was their occupation at the time of the  study? | 6 |
| *4.* | Gender | Was the researcher male or female? | Not reported |
| *5.* | Experience and  training | What experience or training did the researcher  have? | 6 |
| Relationship with participants | | | |
| *6.* | Relationship  established | Was a relationship established prior to study  commencement? | 6 |
| *7.* | Participant knowledge of the interviewer | What did the participants know about the researcher? *E.g. Personal goals, reasons for*  *doing the research* | 6 |
| *8.* | Interviewer characteristics | What characteristics were reported about the interviewer/facilitator? *E.g. Bias, assumptions,*  *reasons and interests in the research topic* | 5-6 |
| **Domain 2: Study design** | | | |
| Theoretical framework | | | |
| *9.* | Methodological orientation and theory | What methodological orientation was stated to underpin the study? *E.g. grounded theory, discourse analysis, ethnography,*  *phenomenology, content analysis* | 10 |
| Participant selection | | | |
| *10.* | Sampling | How were participants selected? *E.g. purposive,*  *convenience, consecutive, snowball* | 5 |
| *11.* | Method of approach | How were participants approached? *E.g. face-*  *to-face, telephone, mail, email* | 5-6 |
| *12.* | Sample size | How many participants were in the study? | 6 |
| *13.* | Non-participation | How many people refused to participate or  dropped out? What were the reasons for this? | 7, Box 1 |
| Setting | | | |
| *14.* | Setting of data  collection | Where was the data collected? *E.g. home, clinic,*  *workplace* | 6 |
| *15.* | Presence of non-  participants | Was anyone else present besides the  participants and researchers? |  |

| *16.* | Description of sample | What are the important characteristics of the  sample? *E.g. demographic data, date* | 7 |
| --- | --- | --- | --- |
| Data collection | | | |
| *17.* | Interview guide | Were questions, prompts, guides provided by  the authors? Was it pilot tested? | 6 |
| *18.* | Repeat interviews | Were repeat interviews carried out? If yes, how  many? | No applicable |
| *19.* | Audio/visual recording | Did the research use audio or visual recording  to collect the data? | 7 |
| *20.* | Field notes | Were field notes made during and/or after the  interview or focus group? | Not applicable |
| *21.* | Duration | What was the duration of the interviews or  focus group? | 6 |
| *22.* | Data saturation | Was data saturation discussed? | 6 |
| *23.* | Transcripts returned | Were transcripts returned to participants for  comment and/or correction? | Not applicable |
| **Domain 3: analysis and findings** | | | |
| Data analysis | | | |
| *24.* | Number of data  coders | How many data coders coded the data? | 8 |
| *25.* | Description of the  coding tree | Did authors provide a description of the coding  tree? | Figure 1 |
| *26.* | Derivation of themes | Were themes identified in advance or derived  from the data? | 7-8 |
| *27.* | Software | What software, if applicable, was used to  manage the data? | 7 |
| *28.* | Participant checking | Did participants provide feedback on the  findings? | Not provided |
| Reporting | | | |
| *29.* | Quotations presented | Were participant quotations presented to illustrate the themes / findings? Was each  quotation identified? *E.g. Participant number* | Table 1, Table 2 ( |
| *30.* | Data and findings  consistent | Was there consistency between the data  presented and the findings? | 17-20 |
| *31.* | Clarity of major  themes | Were major themes clearly presented in the  findings? | 10, Figure 1 |
| *32.* | Clarity of minor  themes | Is there a description of diverse cases or  discussion of minor themes? | Not applicable |
