# Supplementary material for: Stakeholders’ perspectives on barriers and enablers of chronic kidney disease care in Ethiopia: A qualitative study
Source: PLoS One. 2025 Nov 13;20(11):e0336781. doi: 10.1371/journal.pone.0336781 (PMC12614622; doi:10.1371/journal.pone.0336781)
Supplement: S2 Appendix — (DOCX) [file pone.0336781.s004.docx]

**S2 Appendix**: Justification of sample size using malterud’s information power Framework

| Parameter | Application to this study | Implication for sample size adequacy |
| --- | --- | --- |
| Study aim | The study explored stakeholders’ perspectives on barriers and enablers of CKD care in Ethiopia. The focus was specific and clearly defined. | A focused aim requires fewer participants to obtain sufficient information power. |
| Sample specificity | Participants were purposively selected with maximum variation, supplemented by snowball sampling. The sample included diverse stakeholders: general practitioners, nephrologists/internists, dialysis nurses, NCD officers, and programme coordinators, working across different health system levels. | High specificity and variation of the sample increased information power and reduced the need for a very large sample. |
| Use of established theory | Data collection and analysis were guided by the Theoretical Domains Framework (TDF), which structured the coding process. | Use of an established theoretical framework strengthened analytic depth and reduced the need for a larger sample. |
| Quality of dialogue | Semi-structured interviews (35–54 minutes) were conducted by a trained qualitative researcher (DK) in Amharic, ensuring rich, in-depth narratives. Participants had no prior relationship with the interviewer, which facilitated openness. Despite online modality flexible scheduling enhanced dialogue quality. | High-quality dialogue increased information power and supported adequacy of a smaller sample. |
| Analysis strategy | A thematic analysis was conducted, guided by the TDF domains. Data from different stakeholder groups were merged to generate overarching themes. | Merging data across stakeholder groups required diversity in perspectives, which was achieved through purposive and snowball sampling. |
| Sample size | Fifteen participants were interviewed. Recruitment spanned February–December 2024, ensuring inclusion of diverse perspectives despite the armed conflict context. | The final sample size was adequate to address the study aim and capture variation across stakeholder groups. |

CKD: chronic kidney disease se; NCD: non communicable disease; TDF: Theoretical domain framework
