# Supplementary material for: Stakeholders’ perspectives on barriers and enablers of chronic kidney disease care in Ethiopia: A qualitative study
Source: PLoS One. 2025 Nov 13;20(11):e0336781. doi: 10.1371/journal.pone.0336781 (PMC12614622; doi:10.1371/journal.pone.0336781)
Supplement: S3 Appendix — (DOCX) [file pone.0336781.s005.docx]

**S3 Appendix: Interview guide**

Date and time of interview:

Name of interviewee:

| **Date and time of interview:**  **Name of interviewee:** | __________________________________________________________  ___________________________________________________________ |
| --- | --- |

[Introducing study]

Thank you for taking time out of your busy schedule to participate in our research study. This project aims to explore perspectives of health administrators and health care providers on the barriers and facilitators to chronic kidney disease management in Ethiopia. Please feel free to ask us any questions about the study before we begin. Also, please remember that participation in this study is voluntary. If you would like to end the interview at any time, please do not hesitate to do so.

**Demographics and practice information**

| 1. Gender |
| --- |
| 1. GP/Internist/Nephrologist / |
| 1. May I ask your age? |
| 1. How many years have you been working at the current position? |
| 1. Rural/ urban practice location |
| 1. Team or individual practice type |
| 1. Approximate # of patients in practice/ rostered |
| 1. How many patients do you see per week? |

***Health administrators (regional health officer, NCD program coordinators )***

| *No.* | *Questions* | *Comments* |
| --- | --- | --- |
|  | What are the efforts from the government for non-communicable disease (NCD)? Is there a separate Diabetes Mellitus /CKD control program? |  |
|  | What are the current diabetes and kidney diseases related programmatic initiatives by the Government of Ethiopia? |  |
|  | Where are these programs being implemented currently states (referral hospitals, primary hospital, General Hospitals) |  |
|  | What health service standards are currently established and agreed upon by the Ethiopian Ministry of Health for the treatment of CKD, if any?   - Trained staff and guidelines - Equipment, diagnostics, medicines |  |
|  | What are the main challenges for quality CKD management in Ethiopia aand particularly in the Amhara region? How do you think you can overcome the problem? |  |
|  | How is the overall health delivery for providing quality CKD care to the region ? |  |
|  | What do you think will enable better provision of quality CKD management? |  |

**Nephrologist(internist, residents )**

| No. | Questions | Comments |
| --- | --- | --- |
|  | How do you currently manage patients with CKD? |  |
|  | What are the main challenges nephrologists face with the rising burden of CKD? |  |
|  | What are the current referral practices? How are the patients with CKD referred to you i.e., the mechanism of referral? |  |
|  | What is the approximate rate of people who receive a follow-up to the doctor to which they are referred? |  |
|  | What do you think will be the factors that will encourage better CKD management? |  |
|  | In general, do you feel there is a need to change how DM/CKD is currently managed in your facility? |  |
|  | What are the main challenges for CKD management in your facility? |  |
|  | What do you think will be the factors that will encourage better CKD management? Which one is the most important? Why? |  |

**General practitioners**

| No. | Questions | Comments |
| --- | --- | --- |
|  | What are your current duties as a primary health care doctor? |  |
|  | What are the work practices of GPs in relation to CKD management? |  |
|  | Do you get some training in managing CKD management? |  |
|  | What is the mechanism of referral to the next level of care (nephrologists) for CKD complications? |  |
|  | What do you think will be the factors that will encourage better CKD management ? |  |
|  | In general, do you feel there is a need to change how CKD is currently managed in your facility? |  |
|  | What are the main challenges for CKD management in your facility? |  |

**Dialysis nurse**

| No. | Questions | Comments |
| --- | --- | --- |
| 1 | Interview Guide for dialysis nurse |  |
| 2 | Please tell me about the patient population that comes for dialysis for services. |  |
| 3 | What are the barriers that patients face to accessing care here? |  |
| 4 | What are some of the challenges you face in your work here? |  |
| 5 | How does the demand for services here at your dialysis centre compare with the resources available? |  |
| 6 | Do you think there is a need to expand the dialysis services? |  |
| 7 | Is there anything else you would like to add? |  |
| 8 | Interview Guide for dialysis nurse |  |
