# Supplementary material for: Stakeholders’ perspectives on barriers and enablers of chronic kidney disease care in Ethiopia: A qualitative study
Source: PLoS One. 2025 Nov 13;20(11):e0336781. doi: 10.1371/journal.pone.0336781 (PMC12614622; doi:10.1371/journal.pone.0336781)
Supplement: S4 Appendix — (DOCX) [file pone.0336781.s006.docx]

**S4 Appendix:** Description of theoretical domains framework (TDF)

| Domains | *Definition* | *Theoretical constructs represented within each domain* |
| --- | --- | --- |
| Knowledge | *An awareness of the existence of something.* | *• Knowledge (including knowledge of condition/scientific rationale) • Procedural knowledge • Knowledge of task environment* |
| Skills | *An ability or proficiency acquired through practice.* | *• Skills • Skills development • Competence • Ability • Interpersonal skills • Practice • Skill assessment* |
| Social/professional role and identity | *A coherent set of behaviours and displayed personal qualities of an individual in a social or work setting.* | *• Professional identity • Professional role • Social identity • Identity • Professional boundaries • Professional confidence • Group identity • Leadership • Organizational commitment* |
| Beliefs about capabilities | *Acceptance of the truth, reality, or validity about an ability, talent, or facility that a person can put to constructive use.* | *• Self-confidence • Perceived competence • Self-efficacy • Perceived behavioral control • Beliefs • Self-esteem • Empowerment • Professional confidence* |
| Beliefs about consequences | *Acceptance of the truth, reality, or validity about outcomes of a behavior in each situation.* | *• Outcome expectancies • Characteristics of outcome expectancies • Beliefs • Anticipated regret • Consequents* |
| Optimism | *The confidence that things will happen for the best or that desired goals will be attained* | *Optimism*  *Pessimism*  *Unrealistic optimism*  *Identity* |
| Reinforcement | *Increasing the probability of a response by arranging a dependent relationship or contingency, between the response and a given stimulus.* | *• Reward (proximal/distal) valued/not valued (probable/improbable) • Incentives • Punishment • Consequents • Reinforcement • Contingencies • Sanctions* |
| Intentions | *A conscious decision to perform a behavior or a resolve to act in a certain way.* | *• Stability of intentions • Stages of change model • Transtheoretical model/stages of change* |
| Goals | *Mental representation of outcomes or end states that an individual want to achieve.* | *• Goals (distal/proximal) • Goal priority • Goal/target setting • Goals (autonomous/controlled) • Action planning • Implementation intention* |
| Memory, attention and decision processes | *The ability to retain information, focus selectively on aspects of the environment and choose between 2 or more alternatives.* | *• Memory • Attention • Attention control • Decision making • Cognitive overload/tiredness* |
| Environmental context and resources | *Any circumstance of a person’s situation or environment that discourages or encourages the development of skills and abilities, independence, social competence, and adaptive behavior.* | *• Environmental stressors • Resources/material resources • Barriers and facilitators • Organizational culture/climate • Person and environment interaction • Salient event/critical incidents* |
| Social influences | *Those interpersonal processes that can cause individuals to change their thoughts, feelings, or behaviors.* | *• Social pressure • Social norms • Group conformity • Social comparison • Group norms • Social support • Intergroup conflict • Power • Slienation • Modeling* |
| Emotion | *A complex reaction pattern, involving experiential, behavioral, and physiological elements, by which the individual attempts to deal with a personally significant matter or event.* | *• Anxiety • Fear • Affect • Stress • Depression • Positive/negative affect • Burn-out* |
| Behavioral regulation | *Anything aimed at managing or changing objectively observed or measured actions.* | *• Self-monitoring • Breaking habit • Action planning* |

Theoretical Domains Framework (TDF) version 2 domains and constructs [1]. This table summarises the 14 TDF domains, their definitions, and associated theoretical constructs.

Atkins L, Francis J, Islam R, O’Connor D, Patey A, Ivers N, et al. A guide to using the Theoretical Domains Framework of behaviour change to investigate implementation problems. Implementation science. 2017;12:1-18.
